# Supplementary material for: Excessive boredom among adolescents: A comparison between low and high achievers
Source: PLoS One. 2020 Nov 5;15(11):e0241671. doi: 10.1371/journal.pone.0241671 (PMC7644046; doi:10.1371/journal.pone.0241671)
Supplement: S1 Table — Total N = 244; group 1 n = 125; group 2 n = 119. M1: Configural invariance. M2: Metric invariance. M3: Scalar invariance. ** p ≤ .01. (PDF) [file pone.0241671.s001.pdf]

Supporting Information

S1 Table. Chi-Squared Difference Test for the Nested Model Comparison.

Model	χ2 (df)	CFI	RMSEA (90% CI)	SRMR	Model comp	Δχ2 (Δdf)	ΔCFI	ΔRMSEA	ΔSRMR	Decision
**SDQ - Emotional symptoms**										
M1	12.6 (10)	.985	.048	.038†	--	--	--	--	--	--
M2	14.8 (14)	.995	.022	.046	M1	2.21 (4)	.010	-.03	.008	Accept
M3	16.5 (18)	1.000	.000†	.049	M2	1.73 (4)	.005	-.02	.003	Accept
**SDQ - Conduct problems**										
M1	16.9 (10)	.926†	.077	.049†	--	--	--	--	--	--
M2	24.6 (14)	.886	.081	.065	M1	7.68 (4)	-.04	.004	.015	Accept
M3	25.9 (18)	.915	.062†	.065	M2	1.32 (4)	0.03	.019	.001	Accept
**SDQ - Hyperactivity**										
M1	22.3 (10)	.933	.101	.055†	--	--	--	--	--	--
M2	25.1 (14)	.939†	.081†	.063	M1	2.86 (4)	.006	-.020	.008	Accept
M3	40.4 (18)**	.877	.102	.083	M2	15.25 (4)**	-.62	.020	.020	Reject
**SDQ - Peer problems**										
M1	12.2 (10)	.970†	.043†	.041†	--	--	--	--	--	--
M2	26.1 (14**	.830	.086	.068	M1	13.97 (4)**	-.140	.043	.027	Reject
M3	27.7 (18)	.864	.068	.070	M2	1.54 (4)	.034	-.018	.002	Accept

Model	χ2 (df)	CFI	RMSEA (90% CI)	SRMR	Model comp	Δχ2 (Δdf)	ΔCFI	ΔRMSEA	ΔSRMR	Decision
**SDQ - Prosocial behavior**										
M1	13.6 (10)	.977	.055	.038†	--	--	--	--	--	--
M2	17.9 (14)	.975	.048	.055	M1	4.23 (4)	-.001	-.007	.016	Accept
M3	21.3 (18)	.979	.039	.059	M2	3.46 (4)	.003	-.009	.005	Accept
**PANAS - Positive affect**										
M1	129 (70)	.883	.086	.066†	--	--	--	--	--	--
M2	136 (79)	.887†	.080	.075	M1	7.00 (9)	.004	-.006	.009	Accept
M3	147 (88)	.882	.077†	.078	M2	11.35 (9)	.005	-.003	.004	Accept
**PANAS - Negative affect**										
M1	175 (70)	.784†	.114	.082†	--	--	--	--	--	--
M2	217 (79)	.773	.110	.096	M1	16.03 (10)	-.012	-.004	.014	Accept
M3	198 (88)	.774	.104	.098	M2	8.20 (9)	.002	-.006	.002	Accept
**ERQ - Cognitive reappraisal**										
M1	6.82 (4)	.982†	.079	.035†	--	--	--	--	--	--
M2	10.68 (7)	.977	.068†	.057	M1	3.86 (3)	-.005	-.011	.022	Accept
M3	18.49 (10)	.947	.086	.070	M2	7.81 (3)	-.030	.018	.012	Accept
**ERQ - Expressive suppression**										
M1	9.77 (4)	.933†	.112	.043†	--	--	--	--	--	--
M2	13.56 (7)	.924	.090†	.053	M1	3.79 (3)	-.009	-.022	.01	Accept
M3	19.57 (10)	.890	.091	.068	M2	6.01 (3)	-.035	.001	.01	Accept

Model	$\chi2$ (df)	CFI	RMSEA (90% CI)	SRMR	Model comp	$\Delta\chi2$ (Δdf)	ΔCFI	ΔRMSEA	ΔSRMR	Decision
**BFI-2 Neuroticism**										
M1	278 (108)	.694	.119	.099†	--	--	--	--	--	--
M2	287 (119)	.697†	.113	.105	M1	8.98 (11)	.004	-.006	.006	Accept
M3	299 (130)	.696	.108	.107	M2	11.78 (11)	-.001	-.005	.002	Accept
**BFI-2 Conscientiousness**										
M1	242 (108)	.765	.108	.094†	--	--	--	--	--	--
M2	251 (119)	.769	.102	.099	M1	8.86 (11)	.004	-.006	.005	Accept
M3	268 (130)	.758	.100	.102	M2	17.23 (11)	.011	-.002	.004	Accept

Total $N = 244$; group 1 $n = 125$; group 2 $n = 119$. M1: Configural invariance. M2: Metric invariance. M3: Scalar invariance. ** $p \leq .01$.
