# Supplementary material for: Excessive boredom among adolescents: A comparison between low and high achievers
Source: PLoS One. 2020 Nov 5;15(11):e0241671. doi: 10.1371/journal.pone.0241671 (PMC7644046; doi:10.1371/journal.pone.0241671)
Supplement: Attachment — Submitted filename: Response to Reviewers.docx [file pone.0241671.s002.docx]

Author Response PONE-D-20-20833

**Reviewer 1**

The authors ask an interesting and important question about boredom in high versus low achieving students. The sample and analysis procedures were adequate to answer the research question. The results provide further insight into students’ experiences of boredom as a negative and persistent emotion.

**Response.** Thank you for this positive evaluation. We also appreciate your suggestions how to revise the manuscript. In response to your comments, we have made the following changes to the manuscript.

1) “Page 1 “Are undesirable correlates of boredom augmented” – augmented might not be the right word choice. Maybe exacerbated? Or even just simply “worse”.”

**Response.** Thank you for this suggestion. We agree and changed “augmented” to “worse” (p. 3).

2) “In the first paragraph, reduce the use of parentheses and add the i.e., likely over/under challenged to the main sentences because it is important content. Same in later parts of the manuscript. (thus likely over/under-challenged) should be part of the sentence.”

**Response.** We agree and removed all parentheses in this context. As a result, for example, the last section of the first paragraph now reads as follows:

*Or is it the excessive boredom per se that covaries with problematic behavior and personality? To address this question, we systematically compared students who are highly bored and low-achieving, that is, likely over-challenged, and highly bored yet high-achieving, that is, likely under-challenged, in the subject of mathematics.* (p. 3)

3) “The literature review is concise, to the point, and up-to-date. However, it would be helpful to add some more details on the “correlates” of boredom rather than using this as a general descriptor. In particular, what is the current evidence on boredom and behavioral and personality variables? This content should go before “The present study” section rather than having little bits of it in that section (references 23-35).”

**Response.** In response to this helpful suggestion, we added a section ‘Boredom as an unpleasant emotion with undesirable correlates’ and placed it before the section ‘Boredom due to being over- vs. under-challenged.’ In response to reviewer 3 (comment #1), in this section it is also mentioned that a key feature of our study was to assess boredom domain-specifically, while still expecting to replicate general boredom correlate findings reported earlier:

*Boredom, most generally, is described as an unpleasant and distressing experience [22]. There are two widely used scales to measure general trait boredom: The Boredom Proneness Scale [BPS; 23] and the Boredom Susceptibility Scale [BSS; 24]. Research on the BPS has revealed that boredom proneness has multiple undesirable correlates, including alexithymia [25], alienation [26], anger and aggression [27–29], impulsiveness [28,30–32], loneliness [23], narcissism [33], negative affect [34], neuroticism [28,35,36] procrastination [37,38], and unsociability [31]. In turn, low levels of boredom proneness have been shown to be linked with higher levels of conscientiousness, openness to experience [6], and life satisfaction [23]. High scores on the BSS have been reported to be associated with higher levels of motor impulsivity, sensitivity to reward, gambling, alcohol, and smoking [36,39]. Going beyond such general, context-transcending findings, the present study specifically addresses boredom at school, and even more specifically, student experiences of boredom in the subject of mathematics. We thus assess boredom as a trait construct in a domain-specific way.*

*While mathematics boredom has been studied in several recent studies addressing, for example, the control- and value-appraisal antecedents of mathematics boredom [9,40], or boredom-achievement links [41], no study to date seems to have explored whether such domain-specific boredom is also linked with person-level behavioral and personality variables. In other words, it remains open to question if those students who report to experience intense boredom in mathematics only show undesirable levels of structs related to the domain of mathematics (e.g., poor study habits), or if they also show problematic behavior patterns beyond this context (e.g., lower sociability). In line with Bronfenbrenner’s [42] ecological systems theory, we suggest that domain-specific boredom and more general behavioral and personal variables inevitably interact with each other. Thus, the first aim of this study was to replicate prior correlational findings as demonstrated using more general instruments for the assessment of boredom in the subject of mathematics.* (p. 4-5)

4) “Please check the phrase “scholastic boredom” for accuracy. I think you just mean achievement boredom?”

**Response.** Thank you for this suggestion. We agree and changed scholastic boredom to achievement boredom throughout the manuscript.

5) “Only 258 of the original 1404 had high boredom? This seems quite small given the introduction puts the prevalence of boredom as 30-40%. This should be discussed maybe as a limitation or direction for future research differently than it is currently done because of its mismatch with current numbers. Also in the limitations section the n=271. Please check for accuracy between the two sections.”

**Response.** Thank you for raising these points. We would like to argue that quantifying boredom prevalence, and comparing it across studies, is quite tricky: In our introduction, we stated that adolescents are bored 30-40% *of the time* at school; this does not necessarily imply that 30-40% of all students experience high boredom. In our study, we deliberately defined *highly bored* students as those who scored higher than one standard deviation above the sample mean on the AEQ-M boredom scale which uses a Likert-type agreement answer scale, implying that this was an extreme group making up only a small fraction of the total sample.

The *n* of 271 in the limitations was indeed an error – thanks for detecting that. We changed it to the correct number of 256. (p. 20)

6) “The discussion is appropriate and does not over generalize. However, it is a bit short. Can you please add some implications for the control-value theory especially because researchers in the area constantly have to manage the “boredom by over or under challenge” critique. I think this paper helps those researchers say that it doesn’t really matter – boredom is boredom.”

**Response.** Following your suggestion, we changed the corresponding section of the discussion, which now reads as follows (p. 18):

*Moreover, and most importantly, our results suggest that high boredom is associated with similar levels of problematic correlates in low- and high-achieving students. The two groups did not significantly differ in emotional symptoms, conduct problems, hyperactivity, peer problems, prosocial behavior, positive and negative affect, neuroticism, cognitive reappraisal and expressive suppression, neuroticism, and conscientiousness. In line with Pekrun’s [44,45] control-value theory of achievement emotions which posits that boredom can occur either when control is particularly high, or when it is particularly low, we find that both over- and under-challenge can lead to high boredom. Furthermore, irrespective of student’s performance, and hence irrespective of their subjective control in a certain domain, our study demonstrates that high boredom itself is associated with many of these problems. In sum, we propose that one important implication from our findings is that boredom is boredom – irrespective of its antecedents.* (p. 18-19)

# **Reviewer 2**

In the study, pupils in fifth to tenth grades who were very bored and high-achieving in mathematics were compared with students who are very bored and low-achieving in mathematics. Comparisons were made regarding different behavioral and personality variables. Results show that there are no mean-level differences across the two groups for the investigated variables with three exceptions: Conduct problems and expressive suppression were higher for very bored low achievers and positive affect was higher for very bored high achievers. The manuscript is well written; the research questions are interesting; and the methods and data analyses are adequate.

**Response.** Thank you for this positive evaluation. We also appreciate your suggestions how to revise the manuscript. In response to your comments, we have made the following changes to the manuscript.

1) “The use of terms and the categorization of the variables under consideration varies within the manuscript, sometimes considerably. In the abstract, the authors mention that behavior and personality aspects will be considered, but in the manuscript other super-categories are often used (on p. 8: emotional and behavioral problems, positive and negative affect, emotion regulation strategies, personality traits; on p. 13: behavioral and affective variables; Table 3: emotional symptoms). A standardization of categories and terms across the entire manuscript would strengthen the manuscript and help readers to follow the authors.”

**Response.** Thank you for this suggestion. The constructs we address in the present study are organized in a three-level hierarchy; there is the overarching term of “behavioral and personality variables”, and there are second-level concepts such as “emotional and behavioral problems” (assessed here by the SDQ), affectivity (assessed here by the PANAS), “emotion regulation” (assessed here by the ERQ), or “personality traits” (assessed here by the BFQ-2). The SDQ, in turn, contains five subscales (emotional symptoms, conduct problems, hyperactivity, peer problems, and prosocial behavior); the PANAS contains two subscales (positive and negative affect), so does the ERQ (cognitive reappraisal and expressive suppression) and selected two subscales from the BFI-2 (neuroticism and conscientiousness).

We agree that consistency is highly desirable. Thus, we used terms more stringent in the revised version of our manuscript. For example, we made sure that we now consistently use the term “behavioral and personality variables” as overarching term, avoid the second-level terms wherever possible and thus predominantly stick to the construct-level terms, and be fully consistent in their labeling. (Abstract, and e.g. p. 4, p. 6, p. 15)

2) “Moreover, the authors should consider providing a somewhat more detailed justification for the selection of the variables they consider.”

**Response.** Thank you for this suggestion. We agree with your suggestion and added more detailed information to the section ‘The present study’ correspondingly:

*The choice of constructs addressed in the present study was guided by the aim to address the central negative aspects mentioned in the general boredom proneness literature, inasmuch as they seemed relevant in our context. We thus aimed at replicating prior findings on a broad range of correlates of boredom as demonstrated using more general instruments for the assessment of boredom proneness, while assessing boredom specifically with respect to the subject of mathematics. Previous research has shown that boredom is linked with enhanced negative emotions [29], conduct problems [20,27,31], hyperactivity [54], peer problems [26], and lack of prosocial behavior [31]. Therefore, to explore potential undesirable correlates of boredom, we took all subscales of the Strength and Difficulties Questionnaire [SDQ, 55] into account. Furthermore, boredom has been shown to be positively linked with negative affect [56], expressive suppression [57], and neuroticism [36] as well as negatively with positive affect [56], cognitive reappraisal [57], and conscientiousness [6]. We therefore additionally considered general affect as measured with the Positive and Negative Affect Schedule [PANAS; 58], cognitive reappraisal and expressive suppression as measured with the Emotion Regulation Questionnaire [ERQ; 59] and finally, neuroticism and conscientiousness as measured with the Big Five Inventory-2 [BFI-2; 60].* (p. 6-7)

3) “A main problem of the study is the way in which the groups of high achievers and low achievers are defined. With a grading system from 1 to 6, it seems questionable whether students with average grades of 3 and 4 can actually be described as high achievers and low achievers respectively. It would be advantageous to exclude the group whose grade averages are in the middle of the applicable grading scale. It can be assumed that the students with 3s and 4s suffer less from boredom due to being over- (i.e., the students with grade averages of a 4) or under-challenged (i.e., the students with grade averages of a 3) than students who actually perform particularly well or particularly poorly, especially since we know that classroom instruction is normally adapted to the needs of average-performing students. A comparison of the two ‘fringe groups’ (possibly using non-parametric tests) therefore seems a more germane approach to answer the question the authors are posing.”

**Response.** Thank you for this suggestion. We see your point – we had been discussing this extensively among the coauthors while working on this project. Let us share here why we came to the conclusion to define our groups by differentiating grades 1-3 and 4-6, and why we would like to stick to this decision. In the German grading system grades of 1 (official verbal label: “excellent”), 2 (“good”), and 3 (“satisfactory”) can be considered as safe and sound. However, with grade 4, a critical range starts (“sufficient”; a final grade of 4 is one grade away of being in danger of having to repeat the year) and goes onwards to 5 (“ poor”; final-year scores of 5 in two subjects implying having to repeat the grade) and 6 (“insufficient”, a single final-year score of 6 implying having to repeat the grade) ). Therefore, we argue that there is a splitting categorical divide between grades 3 and 4 that justifies dividing students into these two groups.

Still, we value your suggestion and reanalyzed the data leaving out students with grades 3 and 4 (see Table 1 below). The results remain basically unchanged – there were significant differences between the two groups (grades 1-2 and 5-6) for only one variable, similar to the original analysis (grades 1-3 and 4-6) where no single group differences were significant (applying alpha-level adjustments to account for repeated testing, as requested by Reviewer 3, comment #18). However, by eliminating students with grades 3 and 4, we would reduce our sample substantially from 244 to 65 participants. Therefore, we chose to prioritize to report data based on the whole sample of 244 highly bored students. Yet to acknowledge this point of debate, we added the following paragraph to the ‘Limitations’ section:

*First of all, the present study relies on the reasoning that the combination of high boredom with good grades in mathematics implies that those students tend to be bored due to being under-challenged, while the combination of high boredom with poor grades implies that they tend to be over-challenged. It is important to note that this is an assumption, and the classification as over- vs. under-challenge may not have been fully valid for each individual student in the two groups. However, we deliberately chose to assess domain-specific boredom and domain-specific achievement separately, to first identify students with very high boredom, and then classify boredom as likely being due to over- vs. under-challenge based on students’ achievement. While this indirect approach to assess over- and under-challenge may be a point of debate, we also deem more direct self-report assessments (e.g., ‘I am bored because it's too easy’) as psychometrically problematic. Items combining reports of boredom with attributions of boredom are double-barreled and thus ambiguous – it is unclear if students who endorse those items do so because they are bored, or because they find the material easy vs. hard, or because they attribute boredom to over- or under-challenge.* (p. 19-20)

Table 1

*Comparing Analyses of Grades 1-3 vs. 4-6 and Grades 1-2 vs. 5-6*

|  |  | Comparison of Latent Means | | | | | |
| --- | --- | --- | --- | --- | --- | --- | --- |
|  |  | Original Analysis:  Grades 1-3 (*n* = 119) vs. 4-6 (*n* = 125) | | | Alternative Analysis:  Grades 1-2 (*n* = 34) vs. 5-6 (*n* = 31) | | |
| Scale | Construct | β | *p* | *R^2^* | β | *p* | *R^2^* |
| SDQ | Emotional symptoms | -.14 [-.160, .005] | .067 | .02 | -.31 [-.563, .038] | .087 | .09 |
|  | Conduct problems | -.23 [-.326, -.058] | .005 | .05 | -.25 [-.706, .050] | .089 | .06 |
|  | Hyperactivity | -.04 [-.266, .143] | .555 | < .00 | .06 [-.422, .553] | .792 | < .00 |
|  | Peer problems | -.17 [-.174, .013] | .091 | .03 | -.17 [-.248, .090] | .361 | .03 |
|  | Prosocial behavior | .01 [-.089, .105] | .874 | < .00 | .15 [-.114, .385] | .286 | .02 |
| PANAS | Positive affect | .20 [.060, .420] | .009 | .04 | .48 [.349, 1.094] | .000 | .23 |
|  | Negative affect | -.12 [-.457, .046] | .110 | .01 | -.32 [-1.063, .002] | .051 | .10 |
| ERQ | Cognitive reappraisal | .12 [-.055, .331] | .162 | .01 | .17 [-.152, .550] | .266 | .03 |
|  | Expressive suppression | -.19 [-.699, - .014] | .041 | .04 | - .01 [-.137, .132] | .969 | < .00 |
| BFI-2 | Neuroticism | -.11 [-.191, .040] | .199 | .01 | -.21 [-.629, .152] | .231 | .04 |
|  | Conscientiousness | -.02 [-.325, .242] | .774 | < .00 | -.03 [-.713, .573] | .830 | < .00 |

*Note.* Bonferroni adjusted p-value < .005. *R*^2^ = coefficient of determination.

4) “A discussion of the differences between school types would be important. Very bored low achievers in the low-achiever track of secondary school might differ from very bored low achievers in the high-achiever track of secondary school. Differences between the tracks could be analyzed or analyses could be controlled for school type.”

**Response.** Thank you for your suggestion. Generally, we do trust the validity of the AEQ-M scale across school types and propose that if a student reports to be highly bored based on the AEQ items, this corresponds with one and the same psychological-affective experience; irrespective of which school type they attend. Nevertheless, we checked this and reran our analyses while controlling for school type (see Table 2 below). Since the results remain completely unchanged and also because school type is not at the focus of our study, we decided to only briefly mention this in the manuscript:

*It is worth noting that these results proved to be fully robust when entering school type as dummy-coded control variables.* (p. 17)

Table 2

*Comparing Analysis Without vs. With Controlling for School Type*

|  |  | Comparison of Latent Means | | | Comparison of Latent Means While Controlling for School Type | | | |
| --- | --- | --- | --- | --- | --- | --- | --- | --- |
| Scale | Construct | β | *p* | *R^2^* | | β | *p* | *R^2^* |
| SDQ | Emotional symptoms | -.14 [-.160, .005] | .067 | .02 | | - .16 [-.166, -.001] | .047 | .03 |
|  | Conduct problems | -.23 [-.326, -.058] | .005 | .05 | | -.20 [-.310, -.037] | .013 | .04 |
|  | Hyperactivity | -.04 [-.266, .143] | .555 | < .00 | | -.03 [-.246, .161] | .684 | < .00 |
|  | Peer problems | -.17 [-.174, .013] | .091 | .03 | | -.13 [-.160, .031] | .186 | .02 |
|  | Prosocial behavior | .01 [-.089, .105] | .874 | <.00 | | .04 [-.072, .116] | .649 | < .00 |
| PANAS | Positive affect | .20 [.060, .420] | .009 | .04 | | .20 [.050, .429] | .013 | .04 |
|  | Negative affect | -.12 [-.457, .046] | .110 | .01 | | -.11 [-.440, .068] | .151 | .01 |
| ERQ | Cognitive reappraisal | .12 [-.055, .331] | .162 | .01 | | .14 [-.024, .370] | .085 | .02 |
|  | Expressive suppression | -.19 [-.699, - .014] | .041 | .04 | | -.19 [-.689, -.018] | .039 | .04 |
| BFI-2 | Neuroticism | -.11 [-.191, .040] | .199 | .01 | | -.12 [-.203, .042] | .197 | .01 |
|  | Conscientiousness | -.02 [-.325, .242] | .774 | <.00 | | < .00 [-.278, .298] | .944 | < .00 |

*Note.* Bonferroni adjusted p-value < .005. *R*^2^ = coefficient of determination.

5) “At some points in the discussion the conclusions should be softened somewhat. For example, it cannot be clearly concluded from the results that boredom itself is associated with many problems regardless of scholastic performance, or that the situation seems to be even more dramatic for low-achieving students.”

**Response.** Thank you for your suggestion. We agree and softened the text in the discussion section. We completely removed the notion of the situation being “more dramatic” for low-achieving students. In addition, we agree that it is not boredom as such, but only extreme levels thereof, which are associated with many problems (while not making any causal inferences from those correlative patterns). At the corresponding point in the discussion, we now also considered Reviewer 1’s request (comment #5) to discuss our results from the perspective of control-value theory. The revised text reads as follows:

*Moreover, and most importantly, our results suggest that high boredom is associated with similar levels of problematic correlates in low- and high-achieving students. The two groups did not significantly differ in emotional symptoms, conduct problems, hyperactivity, peer problems, prosocial behavior, positive and negative affect, neuroticism, cognitive reappraisal and expressive suppression, neuroticism, and conscientiousness. In line with Pekrun’s [44,45] control-value theory of achievement emotions which posits that boredom can occur either when control is particularly high, or when it is particularly low, we find that both over- and under-challenge can lead to high boredom. Furthermore, irrespective of student’s performance, and hence irrespective of their subjective control in a certain domain, our study demonstrates that high boredom itself is associated with many of these problems. In sum, we propose that one important implication from our findings is that boredom is boredom – irrespective of its antecedents.* (p. 18-19)

6) “Minor issue: On page 10, the authors write that SDQ conduct and peer problems showed low reliabilities (alpha between .47 and .71). The second value does not match the value in Table 2 (and is not small).”

**Response.** Thank you for noticing this mistake. We changed it to the correct value:

*SDQ-conduct and peer problems showed low reliabilities (α between .47 and .53).* (p. 12)

# **Reviewer 3**

“Thank you for offering me the chance to review this paper. Overall, I liked the paper and I think it provides a new element to the research on academic boredom. The analyses are not complex but nonetheless adequate and done in an advanced way (e.g. latent construct correlations). It should be noted that the current study uses cross-sectional data only and that it relies on self-reported data as far as I could see.”

**Response.** Thank you for this positive evaluation. We also appreciate your suggestions how to revise the manuscript. In response to your comments, we have made the following changes to the manuscript.

1) “While boredom was assessed specifically for the math class, the other measures seem to be of a more general nature. It seems like more general personal characteristics are thus inter-mixed with math-specific ones. How does this affect the conclusions that can be drawn?”

**Response.** Thank you for this comment. We deliberately chose to explore links between domain-specific boredom and general behavioral and personality variables. In line with Bronfenbrenner’s (1992) ecological systems theory we propose that domain specific boredom and more general behavioral and personal variables inevitably interact with and influence each other. We argue, though, that it is not a trivial result from our study that prior findings on relations between general boredom proneness as assessed with the BPS (Farmer & Sundberg, 1986) or the BSS (Zuckerman, 1979) and general behavioral and personality variables could be replicated when boredom is operationalized as students’ actual boredom in the context of a specific academic domain such as mathematics. This expands knowledge on boredom and shows that math boredom not only correlates with mathematics-specific behavior (such as commitment to doing mathematics homework, or on-task behavior in math class), but is linked with variables beyond the domain of mathematics, for example, with general emotional symptoms, conduct and peer problems, as well as positive and negative affect, neuroticism, and conscientiousness.

In response to your helpful comment, we added a section ‘Boredom as an unpleasant emotion with undesirable correlates’ and placed it before the section ‘Boredom due to being over- vs. under-challenged.’ In response to Reviewer 1 (comment #3), this section also describes the correlates of boredom in more detail:

*Boredom, most generally, is described as an unpleasant and distressing experience [22]. There are two widely used scales to measure general trait boredom: The Boredom Proneness Scale [BPS; 23] and the Boredom Susceptibility Scale [BSS; 24]. Research on the BPS has revealed that boredom proneness has multiple undesirable correlates, including alexithymia [25], alienation [26], anger and aggression [27–29], impulsiveness [28,30–32], loneliness [23], narcissism [33], negative affect [34], neuroticism [28,35,36] procrastination [37,38], and unsociability [31]. In turn, low levels of boredom proneness have been shown to be linked with higher levels of conscientiousness, openness to experience [6], and life satisfaction [23]. High scores on the BSS have been reported to be associated with higher levels of motor impulsivity, sensitivity to reward, gambling, alcohol, and smoking [36,39]. Going beyond such general, context-transcending findings, the present study specifically addresses boredom at school, and even more specifically, student experiences of boredom in the subject of mathematics. We thus assess boredom as a trait construct in a domain-specific way.*

*While mathematics boredom has been studied in several recent studies addressing, for example, the control- and value-appraisal antecedents of mathematics boredom [9,40], or boredom-achievement links [41], no study to date seems to have explored whether such domain-specific boredom is also linked with person-level behavioral and personality variables. In other words, it remains open to question if those students who report to experience intense boredom in mathematics only show undesirable levels of structs related to the domain of mathematics (e.g., poor study habits), or if they also show problematic behavior patterns beyond this context (e.g., lower sociability). In line with Bronfenbrenner’s [42] ecological systems theory, we suggest that domain-specific boredom and more general behavioral and personal variables inevitably interact with each other. Thus, the first aim of this study was to replicate prior correlational findings as demonstrated using more general instruments for the assessment of boredom in the subject of mathematics.* (p. 4-5)

2) “Can there a stronger case be made regarding the degree to which the two selected groups are actually over/under challenged?” and “How fair is it to consider these groups to be over-/under challenged? Is there something more that can be done to show this?”

**Response.** Thank you for raising this important point. We are convinced that grades 1-3 (i.e. “good grades” in the German school context) in combination with very high boredom values are an indication that these students have at least not been permanently overwhelmed in class; vice versa, we consider grades 4-6 (clearly to be interpreted as “problematic” in the German school context) in combination with very high boredom values an indication that these students tend to be over-challenged. We acknowledge, though, that this is somewhat speculative and that for some students the classification into either the over- vs. underchallenged group might not be perfect. Therefore, we are only cautiously raising the implication of over- vs. under-challenge and mainly report our findings with respect to the students being high versus low achievers. To acknowledge this important point of debate within our study, we added the following section to the ‘Limitations’ section:

*First of all, the present study relies on the reasoning that the combination of high boredom with good grades in mathematics implies that those students tend to be bored due to being under-challenged, while the combination of high boredom with poor grades implies that they tend to be over-challenged. It is important to note that this is an assumption, and the classification as over- vs. under-challenge may not have been fully valid for each individual student in the two groups. However, we deliberately chose to assess domain-specific boredom and domain-specific achievement separately, to first identify students with very high boredom, and then classify boredom as likely being due to over- vs. under-challenge based on students’ achievement. While this indirect approach to assess over- and under-challenge may be a point of debate, we also deem more direct self-report assessments (e.g., ‘I am bored because it's too easy’) as psychometrically problematic. Items combining reports of boredom with attributions of boredom are double-barreled and thus ambiguous – it is unclear if students who endorse those items do so because they are bored, or because they find the material easy vs. hard, or because they attribute boredom to over- or under-challenge. (p. 19-20)*

3) “Are you also referring to undesirable personality traits?”

**Response.** Thank you for pointing this out. Yes, we are referring also to undesirable personality traits and changed the labeling, as requested by Reviewer 2 (comment #1). The text now reads as follows:

*Existing research shows that high achievement boredom is correlated with a range of undesirable behavioral and personality variables and that the main antecedents of boredom are being over- or under-challenged.* (p. 2)

4) “Could replace ‘no study to date…’ with ‘merely knowing that student are highly bored’”

**Response.** Thank you for this suggestion. We agree and changed the first two sentences of the Abstract which now read as follows:

*However, merely knowing that students are highly bored, without taking their achievement level into account, might be insufficient for drawing conclusions about students’ behavior and personality.* (p. 2)

5) “The framing at the start of the paper could be geared somewhat more towards the Edu context.”

**Response.** Thank you for this suggestion. We agree and revised the start of the introduction correspondingly:

*Boredom is one of the most commonly experienced emotions in educational settings [1,2]. Adolescents report being bored 30-40% of the time in school [3,4], but also in their spare time [5]. Highly bored students were shown to avoid schoolwork [6], to have attention problems, and reduced effort, self-regulation, and motivation [7–9]. They were also shown to use less effective learning strategies [2,9]. As a consequence, there is consistent evidence that boredom correlates negatively with academic achievement [9–17]. More generally, high boredom among adolescents has been associated with numerous serious problems like dropping out of school [4,18] or juvenile delinquency [19,20].* (p. 3)

6) “It is not entirely clear what ‘it’ refers to in the 5th sentence (…whether it is similarly…)”

**Response.** Thank you for pointing this out. We changed ‘it’ to ‘boredom’. The sentence now reads as follows:

*However, it is unclear whether boredom is similarly severe when students are bored due to over-challenge and when they are bored due to under-challenge.* (p. 3)

7) “I would suggest summarizing the theoretical and practical relevance explicitly and right away in the first para – ideally beyond ‘this has not been done before’… Also see ‘the present study’.”

**Response.** Thank you for your suggestion. We agree and added the following sentence to the introduction:

*The present study thus seeks to enrich the literature by enhancing our understanding of achievement boredom. Specifically, we add further knowledge about a potential differentiation between boredom due to being over- vs. under-challenged and offer practical implications for teachers, students, and parents.* (p. 3-4)

Moreover, to point out the relevance of our study we revised the end of ‘The present study’:

*Therefore, we formulated the following exploratory research question: Do low-achieving students with high boredom systematically differ in their self-reported behaviors and personality traits from high-achieving students with high boredom? We propose that an answer to this question enhances the scientific understanding of achievement boredom and offers practical implications, especially with respect to potentially dealing differentially with students who are bored due to being over- vs. under-challenged.* (p. 7-8)

8) “p4 second para: I would like to propose to delete statements like ‘this has not been done before’ but rather explain what we can gain from investigating these things.”

**Response.** Thank you for your suggestion. We agree and have deleted the sentence ‘…no study known to us…’ (p. 5) and added actual expected implications from our findings to the introduction section of our revised manuscript (see comment #7).

9) “Why is it important to know whether or not the same correlates exist for the two groups of students?”

**Response.** Thank you for your comment. We believe that *“despite the extensive body of research examining achievement boredom in adolescents, it is still open to question whether experiencing intense boredom is similarly severe when students are low-achieving and when they are high-achieving.”* (p. 7) and that “*add further knowledge about a potential differentiation between boredom due to being over- vs. under-challenged and offer practical implications for teachers, students, and parents.”* (p. 7-8)

10) “Also, I do not see why conscientiousness would protect one from being bored? Conscientiousness could even strengthen feelings of being under-challenged? In sum, I think providing a stronger and more coherent rational for doing this investigation is needed.”

**Response.** Thank you for raising this important point. We argue that it could be hypothesized that high performing students may not suffer as much from undesirable correlates of boredom as low performing students (see e.g. p. 6, “Those factors could protect against the potential undesirable correlates of boredom.“). We think that with all changes made to the introduction based on the reviewers’ suggestions, the rationale for our study is more clearly explained and more coherent now (see e.g. pp. 4, 5, 7).

11) “The effective sample-size is 245 for the analysis of the main research question, this should be stated more explicitly, probably also in the abstract. There, only an N of 1404 students is mentioned describing the entire pool of students, which may raise wrong expectations. Alternatively, a more broad/general research question could be added?”

**Response.** Thank you for this suggestion. It was not our intention to raise wrong expectations. Our analyses consist of two parts, the correlation analyses, where we indeed used the full sample of *N* = 1.404 students, and the follow-up analyses involving only the highly bored students. We explicitly state the sample size for that set of analyses in the abstract:

*In comparing highly bored students (more than one SD above M, n = 258) who were low vs. high achievers (as indicated by the math grade, n = 125 / n = 119) …* (p. 2)

12) “I wonder whether students of 9 year old can reliably answer questionnaires like the ERQ?”

**Response.** We think that the age of our students does not poses a major issue. The ERQ in particular has been validated in France for children aged 8 to 16 years old (Gosling, Noblecourt & Moutier 2018). Furthermore, it is worth noting that there were only five 9-year-olds in our sample.

Gosling, C., Noblecourt, K. & Moutier, S. (2018). The French version of the emotion regulation questionnaire for children and adolescents: Adaptation and validation of the French version. *Enfance*, no 2(2), 291-304. https://www.cairn.info/journal-enfance-2018-2-page-291.htm.

13) “Achievement: I suppose grades were self-reported? Please clarify in the paper.”

**Response.** Yes, the grades were self-reported. We agree that this should be clarified in the paper. We added this information to the manuscript:

*Self-reported math grades from students’ last final report card were used as an indicator of achievement.* (p. 10)

14) “Please explain how the chosen analyses can account for reliabilities low as .47/.53? It would probably have been more straight forward to leave these scales out of the analyses?”

**Response.** The SDQ is a well-established, very widely used instrument, while the internal consistencies for the its subscales have been reported to be relatively low in prior research (e.g. Goodman, 2001). We suppose it would have been problematic to leave subscales out of the analysis when using this well-known instrument. We controlled for measurement error and low reliability by using latent modeling. In response to your comment, we added the first sentence to the manuscript:

*SDQ-hyperactivity and prosocial behavior, ERQ-cognitive reappraisal, and expressive suppression showed borderline-acceptable reliabilities, but SDQ-conduct and peer problems showed low reliabilities (α between .47 and .53). However, earlier studies also documented comparably low internal consistencies for those SDQ subscales when using student ratings [69]. Therefore, this was not a peculiarity of our sample. To circumvent biased results due to scale unreliability, we chose to model all variables as latent constructs using the Lavaan 0.6-5 package [70] employing the full information likelihood method [FIML; 71] for treating missing data, and the MLR estimator (maximum likelihood estimation with robust (Huber-White) standard errors and a scaled test statistic that is (asymptotically) equal to the Yuan-Bentler test statistic).* (p. 12)

Goodman, R. (2001). Psychometric properties of the strengths and difficulties questionnaire. *Journal of the American Academy of Child and Adolescent Psychiatry*, *40*(11), 1337–1345.

15) “Please add the mean levels of boredom in the two groups that are created (or have I overlooked this?).”

**Response.** Thank you for pointing this out. We had indeed missed this and agree that this is of course a helpful statistic to report. We added the mean levels to the last sentence of the “Data Analyses” chapter, which now reads as follows:

*As a result, there were 125 students in the low achievement group (boredom M = 3.98, SD = .53), and 119 students in the high achievement group (boredom M = 3.80, SD = .42).* (p. 14)

16) “Wouldn’t it be more fitting to test interaction terms with low/high achievement and boredom?”

**Response.** Thank you for this question. We agree that this would have been an alternative approach to answer our research question. Our analyses were similar to modeling interaction effects of achievement and boredom on our target outcome variables, just that our approach focused on directly comparing low- vs. high-achieving students who are highly bored. We deliberately chose to do so as we intended to map our results on the widely debated dichotomous conceptualization of boredom due to over- vs. under-challenge in the achievement boredom literature.

17) “In any case: have you considered to correct the alpha levels for repeated testing?”

**Response.** Thank you for this suggestion. In response, we used Bonferroni-corrected alpha levels, and adjusted our results and discussion section accordingly (see e.g. Table 3 and pp. 17, 18-19).

*To account for multiple testing, we used the Bonferroni method to adjust the alpha level to 0.005.* (p. 14)

18) “I think the authors rightly conclude that it is in first place boredom as such rather than the combination with achievement that correlates with problematic student characteristics aso. Therefore, it may be a little overstated (especially given the very low R2s) that the situation is ‘more dramatic’ for low achievers.”

**Response.** Thank you for this comment. We agree and deleted this statement from the discussion section.

19) “Conduct problems had measurement issues, this should be mentioned along with the conclusions. Maybe it should receive a little less attention in the conclusions as well?”

**Response.** Thank you for your suggestion. We agree and fully removed conduct problems from the conclusion as it also turned out not to be significant after alpha level adjustment.
